# Craniofacial Measurements and Indices Trends in Latvian Children Aged 1–15

**DOI:** 10.3390/children11091141

**Published:** 2024-09-20

**Authors:** Arta Grabcika, Dzintra Kazoka, Janis Vetra, Mara Pilmane

**Affiliations:** Department of Morphology, Institute of Anatomy and Anthropology, Rīga Stradiņš University, LV 1010 Riga, Latvia; dzintra.kazoka@rsu.lv (D.K.); janis.vetra@rsu.lv (J.V.); mara.pilmane@rsu.lv (M.P.)

**Keywords:** children, age, head, face, anthropometry, indices

## Abstract

Craniofacial development is a critical aspect of pediatric growth, influencing fields such as physical anthropology, pediatrics, forensic sciences, and clinical practice. Objectives: This study aimed to assess the trends in craniofacial measurements and indices in Latvian children aged 1–15. Methods: The anthropometrical measurements (head circumference, length, width, facial length, and width) were conducted on 375 children according to the guidelines by R. Martin, K. Saller, and J. Prīmanis at the Anthropology laboratory at the Institute of Anatomy and Anthropology, Rīga Stradiņš University. The cephalic index (CI) and the facial or prosopic index (PI) were calculated, allowing for a detailed examination of cranial and facial proportions. The data were analyzed using descriptive and inferential statistics, including the Pearson Chi-square test and the Spearman correlation coefficient. Results: The findings revealed a consistent increase in head circumference with age, with boys generally having slightly larger heads than girls. The boys and girls exhibited growth in head length and width, particularly noticeable between 1 and 2 years of age. Facial length and width increased with age, with boys typically having longer facial lengths and broader faces than girls. The most common cephalic index category is mesocephaly, which accounts for 52.85% of the population, followed by dolichocephaly at 32.94%. Brachycephaly and hyperbrachycephaly are less prevalent, representing 11.36% and 2.86% of the population. Regarding the facial index categories, hypereuryprosopic is the most prevalent, representing 45.54% of the population, closely followed by euryprosopic at 43.74%. Mesoprosopic is significantly less common, representing only 9.95%. The least common categories are leptoprosopic and hyperleptoprosopic, with prevalences of 0.64% and 0.12%, respectively. The indices analysis showed variations across age groups, with dolichocephaly being more common at younger ages and decreasing over time. Brachycephaly and hyperbrachycephaly remained relatively stable or decreased slightly with age, while mesocephaly displayed less variation. The relationship between the FI and CI in younger age groups was typically weak to moderate and inverse, with a significance level of *p* < 0.001 or *p* < 0.01. However, no meaningful correlation was observed in older children aged 14–15. Conclusions: This study provides valuable insights into the craniofacial development of Latvian children, highlighting the measurements, cranial and facial types, and their variations across age groups.

## 1. Introduction

Craniofacial morphological characteristics refer to the structural features of the skull and face, which are influenced by genetic and environmental factors [1,2]. These characteristics vary across ethnic groups, geographic regions, gender, age, and individuals [3]. Comparative studies among major human groups have highlighted similarities and differences [4]. Accurate measurement techniques play a crucial role in ensuring the reliability of collected data. The procedures can also include anthropometric measurements to help identify children’s health issues, evaluate infant growth patterns, and determine overall health. Cephalofacial measurements, a crucial part of anthropometry, provide essential data for studying brain growth and the development of facial structures, forming key indicators for research [5,6]. The critical ratio derived from head measurements involving height and width helps in the international classification of head types into dolichocephalic, brachycephalic, mesocephalic, and hyperbrachycephalic categories [7,8]. Several studies have used cephalometrics to examine the anatomical differences between snore individuals and those with sleep apnea [9]. Infants undergo external influences during this critical period of cranial development, including gravity, orientation, and sleeping position, which passively alter the shape of the skull [10]. Variations in head shape could significantly increase susceptibility to obstructive sleep apnea [11]. Furthermore, it has been demonstrated that heightened spring numbers, forces, and lengths are closely linked to amplified changes in the cephalic index (CI) [12]. A study by Villavisanis et al. found that stronger posterior spring forces, when interacting with a thinner posterior calvarial thickness, significantly predicted more remarkable changes in the CI three months postoperatively [13]. It was observed in patients undergoing spring-mediated cranioplasty for non-syndromic sagittal craniosynostosis.

In a child’s early development, the skull grows rapidly, causing changes in the size and shape of the head. The facial bones, on the other hand, grow more slowly and are mainly responsible for changing the overall shape of the skull throughout a person’s life. This process of bone structure development begins between the 23rd and 26th days of gestation. Additionally, skull sutures start to close at around the age of 22 onward [14]. Craniofacial anthropometry has become essential for genetic counselors to identify abnormal syndromes [15].

Cranial dimensions may vary depending on individuals’ ages, typically peaking between 16 and 23 years, and their genetic expression is affected by age-related factors [16]. A significant correlation exists between the inter-premolar width and the facial index. However, no such correlation exists between the intermolar width and facial index, indicating that the facial type and intermolar width are not closely related [17]. Recognizing craniofacial, racial, and group morphological traits enhances the diagnostic precision for growth and developmental abnormalities in children, guides medical, orthodontic, and orthopedic interventions, informs craniofacial reconstructive procedures, aids forensic and anthropological investigations, and, importantly, offers insights to refine risk management strategies for the development of national and local orthodontic healthcare programs for children [3]. Individuals with Apert’s syndrome often have a condition known as hyperbrachycephaly. Meanwhile, Cohen syndrome typically involves a small head and a variable cephalic index. An abnormal cephalic index could indicate a chromosome anomaly. These cephalometric findings can offer valuable insight when evaluating patients before craniofacial surgery [18]. Regardless of the surgical method used, patients with isolated sagittal synostosis often experience an increased cephalic index after surgery, which usually returns to normal levels [19]. The brain reaches 90% of its adult size by the first year of age, and total development typically concludes by age 7.

The cephalic index (CI) is valuable for surgeons and neurosurgeons to assess pre- and postoperative corrections of skull deformities [20]. The findings from cephalometric analysis have a wide range of applications in fields such as forensic medicine, plastic surgery, oral surgery, pediatrics, and dentistry. These findings are also used for comparative diagnostics between patients and normative populations [21,22]. Understanding these cranial characteristics is crucial for performing zygomatic bone surgeries or palatoplasty procedures. This knowledge is essential for deciding whether to partially remove or increase the bone’s size [23,24].

The leptoprosopic type, characterized by a narrow dental arch and an ogival vault, was notably prevalent among individuals exhibiting maxillary compression and open occlusion syndrome, often linked with oral breathing. Conversely, the euryprosopic type, distinguished by broad dental arches and a flat, expansive palate, was prominently observed in patients with deep bite syndrome [25]. Several studies have demonstrated that children with sickle cell disease tend to have a lower cephalic index compared to comparable normal controls. It suggests sickle cell anemia affects bone structure and growth [26].

In the past century, numerous anthropological studies have documented remarkably swift transformations in head shape, unprecedented in human history. These changes vary across countries, with some experiencing brachycephalization while others exhibit dolichocephalization. For instance, the American population’s cranial morphology has shifted towards increased length and narrowness over the last 150 years [27].

Growth disparities between different age groups result in variations in facial dimensions. Numerous research studies have been conducted on the children’s facial index (FI) [28,29]. The researchers indicated that boys typically exhibit a leptoprosopic (long and narrow) facial form followed by an euryprosopic (short and wide) form. In contrast, girls display an euryprosopic form followed by a leptoprosopic form. These findings are consistent with previous research. The observed sexual dimorphism and notable disparity between boys and girls may be attributed to the influence of the hormone testosterone, known to induce shape changes in the face between the two sexes [30]. It has been observed that specific malocclusions correlate with particular facial types, underscoring clinicians’ importance in recognizing each patient’s facial characteristics. This assessment is crucial for the initial classification, treatment planning to address existing issues, and early prognostication of treatment outcomes. The dolichofacial pattern is characterized by a long, narrow face with weak musculature and frequently crowded dental arches. The brachyfacial pattern is short and wide, with a robust, square mandible and broad dental arches [31]. Gopinath et al. found significant correlations between the facial index, combined mesiodistal distance of the maxillary anterior, and the maxillary left central incisor crown length when estimating stature [32]. Among nasal breathers, there was a higher prevalence of the brachyfacial type, whereas subjects with obstructive nasal breathing exhibited a lower frequency of this facial morphology [33].

Head circumference is used to estimate brain volume, especially in early childhood. Changes in head circumference growth can indicate issues with brain development. Hydrocephalus is a common condition caused by a blocked cerebrospinal fluid flow, with an occurrence rate between 0.8 to 1.1 cases per 1000 live births. Before signs of increased pressure in the brain or macrocephaly, its initial symptoms often include rapid head circumference growth [34]. Studies have shown that prenatal corticosteroid use is linked to a reduced head circumference, weight, and length at birth, regardless of other factors. Additionally, the level of growth restriction is related to the corticosteroid dosage, suggesting a causal relationship [35]. Koshy et al. have shown that head circumference measurements in early childhood are associated with cognitive outcomes [36]. A smaller head circumference at the age of two has been linked to lower cognitive scores at the ages of two and five.

Early detection and timely intervention are vital in addressing growth disorders. Pediatric medical professionals must diligently monitor children’s growth patterns and expeditiously address deviations to ensure effective treatment and support. Significant deviations from the norm can indicate conditions associated with various developmental issues [37]. Crucitti et al.’s study found that the average head size in individuals with autism was not abnormal [38]. However, they did observe distinct patterns. For boys with autism, there was a higher likelihood of extreme head sizes at birth and between 60 and 100 months, with smaller head sizes between 6 and 11 months and larger head sizes between 12 and 17 months. On the other hand, girls with autism were more likely to have extreme head sizes between 36 and 59 months, with a lower likelihood at birth. Microcephaly is present in around 17% of individuals with ataxia-telangiectasia, often associated with reduced occipital-frontal head circumference, indicating brain injury and various neurodevelopmental disorders [39].

Several authors highlight the role of craniofacial measurements in monitoring and assessing the health and development of young individuals [40,41]. Understanding and recognizing specific features important for diagnosing children’s growth, development, and abnormalities is vital, as they guide medical, forensic, and anthropological investigations and are valuable in orthodontic, surgical, and orthopedic procedures [42]. The craniofacial morphology undergoes continual changes throughout an individual’s lifespan, influenced by genetic factors, environmental variables, diseases, and overall health status. Disparities are highly intricate, as each body part has a specific growth rate, time, and expression.

The analysis of anthropometric data related to the head and face in children has been a focal point in several studies highlighting the far-reaching significance of such data in diverse areas, including but not limited to product design and healthcare [43,44]. Understanding the anthropometric aspects is crucial for ensuring the creation of products and healthcare interventions well-suited to children’s unique requirements. Pediatric medical professionals must safeguard the well-being of affected children by timely identifying cranial and facial issues, determining their etiology, and providing effective therapeutic interventions or appropriate referrals [45].

Examining prevalent craniofacial values and indices presents significant potential for improving preventive healthcare for children in Latvia. A comprehensive understanding of the trends and dynamics of craniofacial measurements can play a pivotal role in the routine health assessment of young individuals, facilitating timely intervention and treatment. A more precise depiction of craniofacial measurement changes and developments from ages 1 to 15 can be achieved, enabling the early detection of potential health issues. Establishing a comprehensive database of craniofacial measurements can advance research into different factors influencing craniofacial development, ultimately leading to the evolution of new preventive measures and treatments. A comparative analysis of these measurements between Latvian children and other ethnic groups may yield valuable insights into craniofacial development’s genetic and environmental determinants. The remarkable improvements have transformed the diagnostic and therapeutic approaches, significantly enhancing precision [46]. Moreover, the integration of advanced technologies holds promise for enhancing the accuracy and efficiency of craniofacial measurements. Ultimately, this research could help create standardized growth charts for Latvian children by carefully analyzing measurements.

It is essential to gather information about the trends and dynamics of craniofacial measurements in Latvian children to conduct preventive examinations and regularly assess the health status of young people. The combined null hypothesis allowed us to test multiple aspects of our data within a single framework. We stated that there is no significant difference in head circumference, facial length, and width between boys and girls, no significant variation in the distribution of the CI and FI categories, and no significant correlation between the CI and the FI with age in Latvian children. Related to this, the current study aimed to assess the trends in craniofacial measurements and indices in Latvian children aged 1–15.

## 2. Materials and Methods

### 2.1. Study Design and Participants

The longitudinal study has been taking place since 2005/2006 and will be completed in 2024/2025. In this study, data were obtained from 2020 to 2021. The sample size was calculated based on a 95% confidence interval, a 5% margin of error, and a 50% proportion in population for boys and girls. Considering the above, the required sample size was determined to be at least 385 participants. In our case, the initial sample size was 503 healthy, full-term newborns born between 36–41 weeks of gestation (259–293 days) to healthy mothers (not known to be hypertensive, diabetic, or in any long-term disease or medications) from normal pregnancies, all born at the Riga Maternity Hospital, with each participant being measured once a year. Children with central nervous system disorders, cardiac conditions, endocrinological issues, metabolic abnormalities, congenital malformations, or chronic illnesses were excluded from the study. Twins or multiple pregnancies were not present in this study. Written consent was obtained from the parents of the children involved, confirming their voluntary participation agreement. A formal agreement was also established with the director of the Maternity Hospital, ensuring institutional support and cooperation.

At age one, 128 participants dropped out of the study. In this comprehensive investigation, 375 healthy Latvian children aged 1 to 15 were enrolled in a longitudinal investigation. Participation was voluntary, and all the participants’ parents provided written informed consent. The study was conducted according to the Declaration of Helsinki and approved by the Institutional Ethics Committee (No 2-PĒK-4/597/2022; 14 December 2022).

The protocol for assessing the children’s physical development was carefully crafted to ensure consistency and precision throughout the study [47,48]. The participants were measured annually, coinciding with their birthdays, to capture their growth metrics as they progressed. These measurements took place at the specialized Anthropology laboratory at the Institute of Anatomy and Anthropology, Rīga Stradiņš University. Throughout the study, a reduction in the number of participants was noted, attributable to reasons such as voluntary withdrawal, emigration, and the impact of pandemics.

### 2.2. Measurements of the Head and Face

In this current study, one examiner had two assistants (nurses). One assistant was responsible for supervising the head position, correctly locating landmarks, and making precise measurements. The other assistant was responsible for recording the findings. The primary professional examiner made direct measurements to reduce interobserver bias, so a reliability test was unnecessary. The measurements were used to establish the reference values for different ages. The time required for each child’s measurement was 30 min, except for children aged 1 to 6. The time was adjusted in these cases based on each child’s age and ability to cooperate.

This study used a set of anthropometric instruments (Sieber Hegner & Co., Inc., Zurich, Switzerland), including standard spreading calipers, round-ended calipers, non-stretchable measurement tape, and Vernier calipers. The measurements were conducted according to the guidelines outlined by R. Martin, K. Saller [47], and J. Prīmanis [48]. The landmarks utilized in this study comprised specific points located on the head and face (Table 1).

In the head, the following parameters were measured and noted: head circumference (glabella-opisthocranion-glabella (g-op-g))—around the head’s surface in the plane defined by the glabella (g) and opisthocranion (op) points; head length (glabella-opisthocranion (g-op))—between the glabella point (g) of the forehead and the most posterior point (op) of the backside of the head; and head width (euryon-euryon (eu-eu))—between the most lateral points of the skull.

In the face, the measurements included facial length (nasion-gnathion (n-gn))—between the nasion point at the root of the nose (n) and the chin point (gn); and the facial width (zygion-zygion (zy-zy))—between the right and left zygion landmarks positioned at the most lateral points of the zygomatic arches.

**Figure 1:**
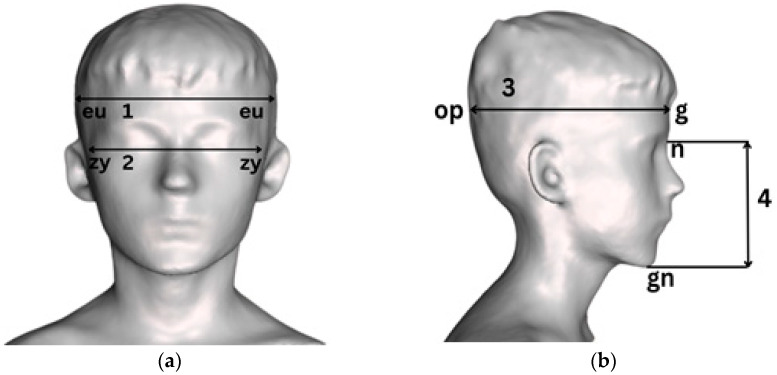
An illustration showing the measuring facial anthropometrics in 3D using the occipital structure sensor: (**a**) Frontal aspect: 1—head width (eu-eu), 2—facial width (zy-zy); (**b**) lateral aspect: 3—head length (g-op), 4—facial length (n-gn). Anthropometric landmarks: eu—eurion; zy—zygion; op—opisthocranion; g—glabella; n—nasion; gn—gnation.

The craniofacial anthropometric data were accurately captured and presented using three-dimensional (3D) imagery with the occipital structure sensor. This technology facilitated an intricate and precise visualization of diverse craniofacial dimensions, offering detailed and accurate 3D renderings for analysis and display (Figure 1). It was necessary to gather current and comprehensive data to create detailed craniofacial profiles.

### 2.3. Statistical Analysis

Data processing involved using Microsoft Office Excel 2021 (16) and IBM SPSS Statistics 28.0 (IBM SPSS, Armonk, NY, USA). The collected data were evenly distributed into age groups, tabulated, and then analyzed using descriptive statistics.

The children were categorized into 15 different age groups for this research. The cephalic index (CI) and facial or prosopic index (FI) were then calculated using internationally recognized anthropometrical standard definitions and descriptions.

The cephalic index (CI) is calculated using the following formula [49]:

$$\mathrm{CI}=\left(\frac{\mathrm{Cephalic}\;\mathrm{Width}}{\mathrm{Cephalic}\;\mathrm{Length}}\right)\times 100$$

The head shape based on the CI was categorized using the classification system developed by Cohen and MacLean [50], and four categories have been established:

- Dolichocephalic: CI ≤ 75.9;
- Mesocephalic: 76.0 ≤ CI ≤ 80.9;
- Brachycephalic: 81.0 ≤ CI ≤ 85.4;
- Hyperbrachycephalic: CI ≥ 85.5.

The facial index (FI) is calculated as follows [39]:

$$\mathrm{FI}=\left(\frac{\mathrm{Nasiomental}\;\mathrm{Length}}{\mathrm{Bizygomatic}\;\mathrm{Width}}\right)\times 100$$

According to Martin–Saller’s scale [37], this index was classified into five groups:

- Hypereuryprosopic (very broad face): PI ≤ 79.9;
- Euryprosopic (broad face): 80.0 ≤ PI ≤ 84.9;
- Mesoprosopic (round face): 85.0 ≤ PI ≤ 89.9;
- Leptoprosopic (long face): 90.0 ≤ PI ≤ 94.9;
- Hyperleptoprosopic (very long face): PI ≥ 95.0.

The mean values, standard deviations, and frequency distributions were used to analyze the continuous and categorical variables. This study applied inferential statistics to discern the disparity between the facial and cephalic indices. The distribution of the cephalic and facial index categories among different age groups was illustrated using proportions and percentages. 

The Pearson Chi-square test was employed for proportion analysis, while the correlation analysis between the facial and cephalic indices was evaluated using the Spearman correlation coefficient.

## 3. Results

In this longitudinal study, we conducted an anthropometric analysis of 375 Latvian children, comprising 183 boys and 192 girls. The age distribution of the participants is presented in Table 2.

The results reveal interesting patterns in craniofacial values, with distinct differences observed between the genders and various age groups.

### 3.1. Head Circumference

In a recent study, the average head circumference for boys was 53.24 cm, whereas for girls, it was 52.21 cm. The research data also revealed that the smallest head circumference was observed at the age of 1, while the largest was at the age of 15. Additionally, the mean head circumference for boys varied from 47.63 cm at age 1 to 56.99 cm at age 15. For girls, the mean head circumference ranged from 46.26 cm at age 1 to 55.26 cm at ages 14 and 15 (Table 3).

### 3.2. Head Length, Width, and Cephalic Index (CI)

The mean head length for all boys was 18.40 cm for boys and 17.70 cm for all girls. The average head length in boys ranged from 16.38 cm to 19.52 cm, but in girls, it ranged from 15.59 cm to 18.66 cm. The average head width was 14.22 cm for all boys and 13.69 cm for all girls. Head width ranged from 12.73 cm to 15.12 cm in boys and 12.11 cm to 14.44 cm in girls.

The mean cephalic index (CI) value in the all-children sample aged 1 to 15 years was 77.36. The average CI was 77.33 for all boys and 77.39 for all girls. The analysis of head shape indicated that the most common type was mesocephalic. In different age groups, the average cephalic index ranged from a minimum of 76.57 to a maximum of 77.77 in boys and from a minimum of 76.34 to 78.12 in girls (Table 4).

### 3.3. Facial Length, Width, and Facial Index (FI)

The mean facial length among boys was 9.85 cm, whereas the average for girls was 9.52 cm. At one year, boys exhibited an average facial length of 7.52 cm, whereas girls had an average of 7.15 cm. Moreover, the mean facial width was 12.26 cm for boys and 11.85 cm for girls. Throughout the first year of their lives, boys typically had an average facial width of 10.77 cm, while the girls’ average facial width was 10.15 cm. As they reached 15, the boys’ mean facial width expanded to 13.81 cm, while for the girls, it increased to 13.11 cm. 

The average facial index (FI) was 80.22 for boys and 80.17 for girls. In the study group of children, it was observed that boys had a minimum FI of 69.82, whereas girls had a minimum FI of 70.44. Conversely, the maximum FI was 82.26 for boys and 82.65 for girls. It is important to note that the research data highlighted a significant prevalence of hyperleptoprosopic facial features within the surveyed population, detailed in Table 5.

### 3.4. Head Shapes

A study of head shape types in children found that dolichocephaly, characterized by a long and narrow head, was most prevalent at one year, with 134 children showing this head shape. This number decreased as the children grew older, with only 11 exhibiting dolichocephaly by age 15. Mesocephaly, characterized by a head shape that is neither too long nor too short, showed relatively high prevalence across various age groups. The highest count of 175 children with mesocephaly was observed at 1 year of age, and this number decreased to 17 by the age of 15. Brachycephaly, characterized by a short and broad head shape, showed lower prevalence than dolichocephaly and mesocephaly. The highest count of 49 children exhibiting brachycephaly was observed at 1 year of age, and by the age of 15, this number decreased to 4. Hyperbrachycephaly, which is characterized by an extremely short and broad head shape, showed the lowest prevalence among all head shape types. The highest count was observed in 17 children at 1 year of age, and this number steadily decreased as the children grew older, with no children exhibiting hyperbrachycephaly by the age of 15 (Table 6).

**Figure 2:**
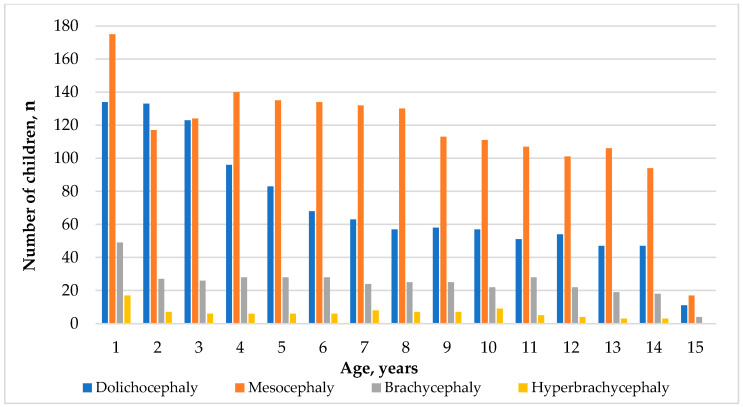
The distribution of head shape types among children of different ages.

Figure 2 illustrates the cephalic index (CI) distribution, representing different head shapes in individuals aged 1 to 15. For every age year, the colored bars represent the number of individuals within each cephalic index category. Figure 2 depicts that dolichocephaly exhibits higher numbers in the early years, particularly at ages 1 and 2, gradually decreasing over time. Regarding mesocephaly, there is a noticeable peak at age 1, and after that, the number of mesocephaly cases gradually reduces but remains relatively stable across the ages. Brachycephaly also shows higher numbers in the early years, decreasing as age increases. The highest number of hyperbrachycephaly cases is around age 1. Later, the number of cases decreases with age, with some fluctuations. Hyperbrachycephaly remains significantly lower throughout the age range than dolichocephaly, mesocephaly, and brachycephaly.

### 3.5. Face Shapes

The distribution of different face shapes across various age groups is fascinating. The hypereuryprosopic face shape, distinguished by broad facial features, is most commonly seen at the age of one, with 240 individuals displaying this characteristic. However, this number gradually decreases to only four individuals by the time they reach the age of 15. On the other hand, the euryprosopic face shape, which is also broad but to a lesser extent than hypereuryprosopic, exhibits variability across the age groups. The highest count of individuals with this face shape, 130, was observed at age 7. Meanwhile, the mesoprosopic face shape, known for its balanced facial proportions, shows relatively stable numbers across the age groups, ranging from 9 to 33 individuals. The leptoprosopic face shape, characterized by narrow facial features, is not very prevalent in early childhood. Still, it gradually increases to four individuals at the age of nine before decreasing once more. Lastly, the hyperleptoprosopic face shape, an extreme form of narrow facial features, is rarely observed across all age groups, with only the occasional presence of one individual at various ages, as indicated in Table 7.

**Figure 3:**
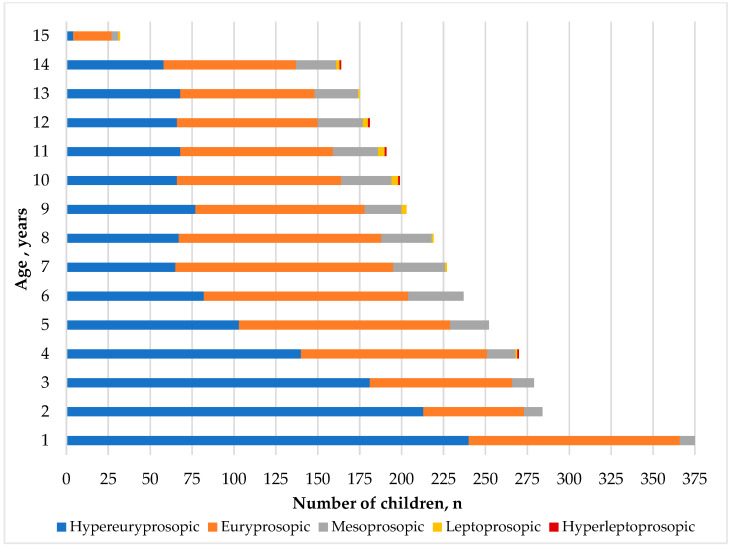
The distribution of facial shape types among children of different ages.

Figure 3 depicts the distribution of five distinct face shape classifications across various age groups of children. The hyperleptoprosopic face shape predominates at ages 1, 2, and 3, with its prevalence diminishing as age advances. Conversely, the prevalence of the euryprosopic face shape escalates, reaching its peak at age 7 before declining by age 15. The mesoprosopic face shape demonstrates a general increase until age 6, followed by marginal fluctuations but an overall decline by age 15. The leptoprosopic face shape becomes evident at age 4, with its prevalence surging at ages 10 and 11 before receding by age 15. Notably, the hyperleptoprosopic face shape only manifests at age 4 and later across various age intervals (10, 11, 12, and 14).

### 3.6. Relationship between Facial Index (FI) and Cephalic Index (CI)

We explored the correlation between the facial index (FI) and the cephalic index (CI) to identify potential relationships. The correlation coefficient was used to measure the strength of the relationship between these two variables. Table 8 displays the correlation values between the FI and CI for different age groups. 

A negative correlation indicates that the FI decreases as the CI increases. Our study found a negative correlation between the CI and FI at ages 1–15. For the youngest age group (1 year), the correlation is weak (r_s_ = −0.250, *p* < 0.001), indicating a statistically significant but weak inverse relationship between the facial and cephalic indices. This pattern of a weak negative correlation continues through the other age groups, with slight variations in strength, such as a moderately negative correlation observed in the 3-year (r_s_ = −0.306, *p* < 0.001) and 5-year (r_s_ = −0.331, *p* < 0.001) age groups.

Significant correlations (*p* < 0.001 or *p* < 0.01) are consistently observed across most age groups, reinforcing the reliability of the observed weak to moderate correlations. However, for ages 14 and 15, the correlation does not appear statistically significant, indicating that the relationship between the facial and cephalic indices in these age groups is not meaningful.

Table 9 presents valuable descriptive statistics insights, including the means and standard deviations, to enhance our understanding of the data distribution and variability.

The mean facial index generally increases with age, starting from 70.13 ± 0.38 in 1-year-olds and reaching 82.28 ± 0.40 by age 15. It suggests a trend toward a higher facial index as children age, with relatively minor variations within each age group, as the standard deviations indicate.

The mean cephalic index remains relatively stable across the age groups, with values ranging from 76.49 ± 0.53 to 77.91 ± 0.60. There is no clear upward or downward trend, indicating that the cephalic index is consistent throughout the different ages studied, with slight fluctuations in the mean values and standard deviations.

**Figure 4:**
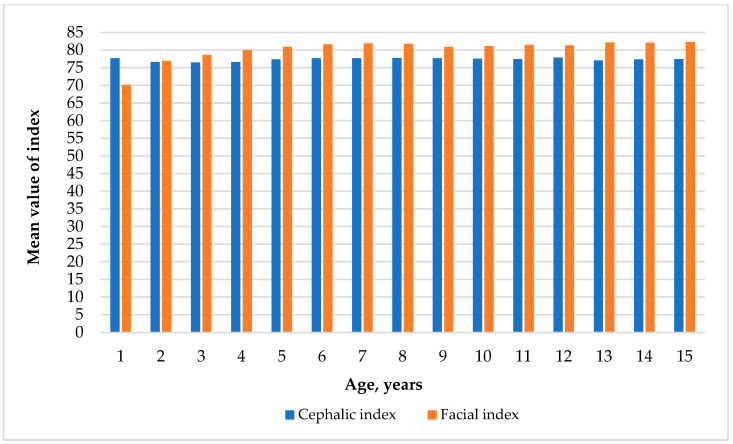
Changes in cephalic and facial indices at different ages.

Figure 4 presents the changes in the mean values of the facial and cephalic indices in different age groups.

### 3.7. Distribution of Facial Types across Different Cephalic Indices (CI)

Table 10 details the analysis of the distribution of facial types across different cephalic index (CI) categories in children aged 1 to 15. In the sample set, hypereuryprosopic face shapes are most commonly found in the mesocephalic group, followed by the dolichocephalic group, the brachycephalic group, and the hyperbrachycephalic group. In the range of measurement periods for children aged 1–15, hypereuryprosopic face shapes constituted 45.54%, with 1496 cases identified.

The prevalence of euryprosopic and mesoprosopic face shapes is highest in the mesocephalic group, followed by the dolichocephalic, brachycephalic, and hyperbrachycephalic groups. Euryprosopic face shapes comprise 43.74% of the sample, with 1437 instances, while mesoprosopic face shapes account for 9.95%, with 327 cases identified.

Leptoprosopic face shapes are less represented among the measured children, primarily in the mesocephalic group, followed by the dolichocephalic, brachycephalic, and hyperbrachycephalic groups.

Hyperleptoprosopic face shapes are the least common. They are mainly found in the dolichocephalic and mesocephalic groups, with some representation in the brachycephalic group. The hyperbrachycephalic group did not exhibit any of these face shapes.

The Chi-square test results demonstrate a statistically significant association between the FI and CI categories (χ^2^ = 89.90, *p* < 0.001). This outcome indicates specific FIs exhibiting a greater propensity to associate with certain CI categories, surpassing the levels anticipated by random occurrence alone.

## 4. Discussion

Anthropometric parameters are crucial in tracking children’s growth and development, offering essential insights into their overall health. Healthcare providers can effectively identify deviations from typical development by establishing normative craniofacial values and indices. Understanding the specific craniofacial characteristics prevalent in the Latvian population is pivotal in devising personalized plans tailored to each child’s unique needs, thereby optimizing the effectiveness of treatments. It enables the early detection of craniofacial trends, facilitating timely interventions and improved outcomes. Various authors emphasize the significance of monitoring changes in head shapes with age in children, as it provides valuable insights into their overall health [51,52]. A clinical report by Dias et al. comprehensively explains the characteristic head shape changes associated with primary craniosynostoses and deformations [53]. The report underscores the critical importance of pediatric care providers being able to adeptly identify children with abnormal head shapes resulting from synostosis and deformational processes. This recognition is vital for early intervention and appropriate medical management. In a study by Fu et al., the focus was on examining the developmental patterns of the head and face in Chinese children [54]. This investigation utilized conventional measurement methods and advanced 3D scanning technology. The authors focused on documenting six specific dimensions of the head and face, including head circumference, head length, head width, forehead width, face height, and morphological face height. The study’s findings revealed a consistent and ongoing increase in all measured head and face dimensions in children aged between 5 and 12.

The craniofacial tendencies and indices in children aged 1–15 can exhibit different variations due to many conditions and factors. Regular head circumference measurements are also indispensable in pediatric healthcare as they help monitor a child’s brain growth and development. Abnormal head growth patterns can indicate conditions such as hydrocephalus, microcephaly, or developmental delays, allowing for early diagnosis and intervention [55,56]. Additionally, tracking the head circumference over time provides valuable data for comparing individual growth against standardized growth charts, ensuring that any deviations are promptly addressed [57]. In this study, the girls and boys displayed a growing trend in head circumference as they got older. Boys typically had slightly larger head circumferences than girls at every age. The disparity in head circumference between boys and girls grew as they aged. Over time, there was a gradual increase in head circumference, with an average annual increase of around 0.78 cm. The increase in head circumference from age 1 to 15 is gradual, with an average increase of around 0.7 cm per year. As the head circumference increases, the head’s length also shows a corresponding increase across all age groups. This observation indicates a positive tendency between head circumference and head length. Similar trends were observed in head width, facial length, and facial width, which generally increased in line with the head circumference.

Icelandic children’s head circumferences were considerably more significant than the WHO’s growth standard, with an overall mean z-score of 1.30 [58]. In our research, the head circumference was also slightly more prominent than the WHO’s growth standard, with a mean z-score of 0.5 to 1.2 between the ages of 1 and 4. In boys, the head length and head width rose between the ages of 1 and 2, with a difference of 0.69 cm and 0.34 cm, respectively. Similarly, girls showed the most significant increase in head length and width between the ages of 1 and 2, with a difference of 0.93 cm and 0.55 cm. We observed a positive trend between the maximum head length and head width, meaning that as the head length increased, the head width also increased in all age groups.

Our data present a significant increase in mesocephalic and dolichocephalic head shapes and a substantial decrease in both sexes’ brachycephalic and hyperbrachycephalic head shapes. Dolichocephaly (long-headedness) appeared to decrease as age increased, with the highest counts at younger ages (1–3 years) and significantly reduced counts as age progressed.

A study of preterm infants born before 32 weeks in North Carolina found that 54% of these infants developed dolichocephaly and received physical therapy [59]. Additionally, it has been observed that this type of head shape in preterm infants may increase the chances of needing physical therapy after being discharged from the hospital. It highlights the importance of addressing dolichocephaly in premature infants with appropriate seriousness despite potential improvements over time [60].

Khatun reported a mean cephalic index (CI) of 75.99 ± 4.97 [61]. This value contrasts with the mean CI observed in several other studies, which ranged from 79.38 to 83.70. However, in our study, the mean CI was 77.36. In a study in Nigeria, the dominant type of head shape was mesocephalic (78.6%) [62]. These findings are specific to Nigerian children and may not be relevant to Latvian children. Banu et al. [3] demonstrated that based on the CI values, the predominant cranial type in children is mesocephalic (62.39%), followed by the dolichocephalic (25.21%) and brachycephalic (12.4%) types. These researchers showed that the dolichocephalic type is more prevalent in boys, while the brachycephalic type is encountered similarly in both sexes. Our study found that the predominant cranial type in children was also mesocephalic (52.85%), with the dolichocephalic type (32.94%) and brachycephalic type (11.36%) following. This comparison highlights that while mesocephalic is the most common cranial type, there are differences in the proportions of each cranial type between the two populations.

In this study, mesocephaly, which refers to having a medium-sized head, showed some variation across ages but remained relatively stable overall. The counts were slightly higher in years 4–9. Brachycephaly, or having a short head, exhibited some variability across ages but generally had lower counts than dolichocephaly and mesocephaly. The counts for brachycephaly remained relatively stable across ages 1–12. Hyperbrachycephaly, which indicates very short-headedness, showed low counts overall, and these counts decreased as age increased, eventually dropping to zero by age 15.

The craniofacial complex undergoes distinct changes at different developmental stages. Facial length increases significantly during childhood and adolescence, with notable growth spurts around puberty [63]. In our study, both girls and boys show a trend of increasing facial length as they age. However, boys tend to have slightly longer facial lengths compared to girls across all age groups. This pattern continues across subsequent years, with both genders experiencing growth in facial length.

Different components of the face grow at different rates [64]. Our findings show that facial width increases with age in both girls and boys. This pattern also continues across subsequent years, with both sexes experiencing growth of this value. By 15 years of age, boys had a mean facial width of 13.81 cm, whereas girls had a mean facial width of 13.11 cm. There was a gradual and consistent increase in facial width as children progressed through adolescence, with boys generally exhibiting broader faces than girls. From 1 to 15 years of age, a positive tendency was observed between facial length and width.

Throughout the study, it was consistently noted that as the facial width increased, the head length decreased across all age groups. However, a positive trend was observed only at ages 7, 12, 13, and 14. Similarly, a positive trend was observed between facial length and head width in all age groups.

An index comprises numerical values describing a population’s relative status on a graduated scale with definite upper and lower limits, which is designed to permit comparisons with other populations classified by the same criteria and methods [65,66]. Precise evaluation of orthodontic treatment requires using accurate and objective measures or indices. These measures consider dental health, aesthetics, and patient satisfaction to determine the urgency and necessity of orthodontic intervention. They are essential in guiding clinicians to make well-informed decisions and ensure that each patient receives personalized treatment. Continuous refinement and development of these measures are crucial for advancing the quality of orthodontic care. In orthodontics, it is important to emphasize the significance of the facial skeletal parameters in the perception of beauty and attractiveness. It is essential to obtain the impact of skeletal growth and development of the upper and lower jaws on the characteristics of the nasal profile. The study by Jankowska et al. involved 386 Polish orthodontic patients aged 9–25, and various nasal and craniofacial measurements were taken and analyzed [67]. The findings showed that the nasal parameters significantly change with age and that there is a notable relationship between nasal morphology and sagittal jaw configuration. Osak et al. investigated the connection between nasal profile structure and various malocclusions, emphasizing the significance of incorporating nasal morphology into orthodontic diagnostic procedures and treatment planning [68].

The facial index (FI) categories are essential for predicting the need for orthognathic surgery. Patients with long faces are the most likely to require surgery, followed by those with short faces and those with average facial proportions [69].

The differences in findings may be due to a combination of ethnic factors and environmental influences [70]. Yesmin et al. performed a study to determine the FI among the Malay population [71]. The researchers discovered that the most common facial type among their population was mesoprosopic. Similarly, Banu et al.’s research identified mesoprosopic as the prevailing facial type (66.24%), followed by the leptoprosopic (26.92%) and euryprosopic (6.84%) types [3]. The study by Asiri et al. showed that the hyperleptoprosopic type of face was most common among children [72]. The FI in the studied groups by Shah et al. was distributed mainly into hypereuryprosopic, followed by euryprosopic types of the face [73]. Our study, however, found that the predominant facial type was hypereuryprosopic (54.54%), with the euryprosopic (43.74%) and mesoprosopic (43.74%) types also being prominent. It indicates that while mesoprosopic was the most common facial type in Banu et al.’s [3] and Yesmin et al.’s [71] studies, our study identified hypereuryprosopic as the leading type, with the euryprosopic and mesoprosopic types also being significant.

In this study, the frequency of individuals with a hypereuryprosopic facial type significantly decreased as age increased. The highest frequency was observed in the 1-year age group, followed by a sharp decline in subsequent years, with very few individuals in the 15-year age group. The euryprosopic facial type remained relatively stable across different age groups, with a slight decrease in older age groups. Mesoprosopic individuals also showed a relatively stable frequency across different age groups, with a slight decline in older age groups, although the frequency was lower compared to the euryprosopic individuals. The leptoprosopic facial type had a consistently low frequency across all age groups, with a slight increase in representation in older age groups. The overall frequency remained relatively low. The hyperleptoprosopic category had an extremely low frequency across all age groups. The differences in the FI values observed between these groups in the above parameters were statistically significant.

The combined null hypothesis helped us to test different aspects of our data and provided a comprehensive understanding of craniofacial development trends. This study found strong evidence to reject the combined null hypothesis, suggesting no significant differences in head circumference, facial length, and width between boys and girls. Our results indicate measurable differences and relationships among these variables. The research findings revealed noteworthy disparities and connections in various aspects. These included substantial variances in head circumference, facial length, and width between boys and girls, notable fluctuations in the prevalence of the cranial index (CI) and facial index (FI) categories across different age groups, and significant correlations between the CI and FI. 

Our study outcomes are valuable for pediatricians in monitoring and assessing cranial and facial development in children. Furthermore, they can contribute significantly to the field of anthropometry by providing updated growth standards and reference data for various populations. The acquired information can be instrumental in revising educational materials for medical and healthcare specialists, ensuring access to the latest craniofacial data of children in Latvia. Additionally, policymakers can utilize this information to formulate guidelines and programs to monitor and promote healthy growth in children. Manufacturers of children’s products can also benefit from this data by enhancing the design of better-fitting and safer products.

While this current study offers valuable insights into craniofacial development among Latvian children, it is crucial to consider potential bias and limitations. Our study faced several potential biases that could impact the validity and generalizability of the findings. Selection bias arises when the participants who remained in the study differed systematically from those who dropped out. In our case, voluntary attrition and migration during the study period, mainly due to the COVID-19 pandemic, may have led to a non-representative sample. Those who continued participating might have different characteristics than those who left, potentially skewing the results. Smaller sample sizes within specific age groups can lead to less reliable results. These smaller groups are more susceptible to random variations, which can affect the accuracy of our findings. Additionally, a limited number of 15-year-old participants, partly due to pandemic-related measurement constraints, may not accurately represent the broader population. Smaller sample bias within specific age groups can lead to less reliable results. The sizes of smaller groups are more susceptible to random variations, which can affect the accuracy of our findings. Additionally, a limited number of 15-year-old participants, partly due to pandemic-related measurement constraints, may not accurately represent the broader population. The pandemic also introduced measurement bias, affecting our ability to collect data, especially from older age groups. It led to potential gaps or inconsistencies in the findings. Comparing our results with other anthropometric studies was difficult due to the differences in age group structures and the use of mean values for the entire sample. Our findings are specific to Latvian children and may not apply to children from other ethnic or geographical backgrounds. It limits the broader applicability of the results. 

Future research should incorporate these aspects to provide a more comprehensive view of our data on craniofacial growth patterns.

## 5. Conclusions

This study provides valuable insights into the craniofacial measurements and indices trends of Latvian children aged 1–15. The boys and girls exhibit an increase in head circumference, head length, head width, facial length, and width with age. The period between 1 and 7 years shows significant changes, emphasizing the crucial importance of this time for craniofacial development. 

In this study, the most common is mesocephaly, which accounts for 52.85% of the population, followed by dolichocephaly at 32.94%. Brachycephaly and hyperbrachycephaly are less prevalent, representing 11.36% and 2.86% of the population. Hypereuryprosopic (45.54%) and euryprosopic (43.74%) are the most prevalent facial index categories, while mesoprosopic (9.95%), leptoprosopic (0.64%), and hyperleptoprosopic (0.12%) are less common.

The notable relationship between facial and cephalic index categories implies a connection, indicating that the characteristics of the face can influence the overall dimensions and proportions of the head, thereby affecting the cephalic index. 

The outcomes of our findings could play a crucial role in shaping policies for the health of Latvian children and developing preventive strategies.

## Figures and Tables

**Table 1:** Definitions and locations of used anthropometric landmarks.

Definition ^1^	Location
Euryon	The most lateral point of the head on the lateral sides of the parietals
Glabella	Most forward-projecting point of the forehead in the midline of the supraorbital ridges
Gnathion	In the midline, the lowest point on the lower border of the chin
Nasion	The point in the midline of both the nasal root and the nasofrontal suture
Opisthocranion	The most posterior point on the protuberance of the occipital bone in the mid-sagittal plane
Zygion	The most lateral point of each zygomatic arch

^1^ Abbreviations: eu—eurion; g—glabella; gn—gnation; n—nasion; op—opisthocranion; zy—zygion.

**Table 2:** Frequency and percentage of children by age in each group.

Age, Years	Boys	Girls
	Frequency, %	Frequency, %
1	183 (48.80)	192 (51.20)
2	138 (48.59)	146 (51.41)
3	135 (48.39)	144 (51.61)
4	128 (47.41)	142 (52.59)
5	116 (46.03)	136 (53.97)
6	110 (46.41)	127 (53.59)
7	104 (45.81)	123 (54.19)
8	100 (45.66)	119 (54.34)
9	91 (44.83)	112 (55.17)
10	91 (45.73)	108 (54.27)
11	87 (45.55)	104 (54.45)
12	81 (44.75)	100 (55.25)
13	80 (45.71)	95 (54.29)
14	76 (46.63)	87 (53.37)
15	15 (46.88)	17 (53.13)

**Table 3:** Means and standard deviations of head circumference for children of different ages.

Age, Years	Boys Head Circumference, cm	Girls Head Circumference, cm	*p*-Value ^1^
1	47.63 ± 0.31	46.26 ± 0.31	<0.001 ***
2	49.90 ± 0.43	48.65 ± 0.26	<0.001 ***
3	51.00 ± 0.42	49.81 ± 0.29	<0.001 ***
4	51.56 ± 0.39	50.49 ± 0.30	<0.001 ***
5	52.10 ± 0.36	51.03 ± 0.31	<0.001 ***
6	52.68 ± 0.40	51.67 ± 0.30	<0.001 ***
7	53.24 ± 0.44	52.08 ± 0.33	<0.001 ***
8	53.67 ± 0.44	52.64 ± 0.28	<0.001 ***
9	54.00 ± 0.44	53.12 ± 0.31	<0.001 ***
10	54.41 ± 0.43	53.57 ± 0.34	<0.001 ***
11	54.58 ± 0.37	53.97 ± 0.34	<0.001 ***
12	54.83 ± 0.38	54.34 ± 0.38	<0.001 ***
13	55.71 ± 0.43	55.01 ± 0.37	<0.001 ***
14	56.33 ± 0.93	55.26 ± 0.40	<0.001 ***
15	56.99 ± 0.41	55.26 ± 0.41	<0.022 *

^1^ * *p* < 0.05; *** *p* < 0.001 statistical significance (independent sample test *t*).

**Table 4:** Characteristics of head length, head width, and cephalic index variations for children of different ages.

Age, Years	Gender	Head Length, cm ^1^		Head Width, cm ^1^		Cephalic Index, %
		M ± SD	Min–Max	M ± SD	Min–Max	
1	Boys	16.38 ± 0.52	15.08–17.92	12.73 ± 0.48	11.42–14.08	77.72
	Girls	15.59 ± 0.58	14.12–16.98	12.11 ± 0.47	10.93–13.67	77.68
2	Boys	17.07 ± 0.41	15.99–18.21	13.07 ± 0.51	11.59–14.31	76.57
	Girls	16.52 ± 0.61	14.79–18.01	12.66 ± 0.53	11.17–14.23	76.65
3	Boys	17.62 ± 0.49	16.31–18.89	13.42 ± 0.57	11.83–14.87	76.63
	Girls	16.95 ± 0.60	15.40–18,50	12.94 ± 0.53	11.37–14.43	76.34
4	Boys	17.92 ± 0.57	16.53–19.57	13.73 ± 0.48	12.32–15.08	76.61
	Girls	17.22 ± 0.59	15.51–18.99	13.19 ± 0.48	11.92–14.58	76.62
5	Boys	18.10 ± 0.58	16.72–19.88	14.01 ± 0.42	12.78–15.32	77.39
	Girls	17.43 ± 0.59	15.71–19.39	13.41 ± 0.45	12.25–14.85	77.40
6	Boys	18.27 ± 0.60	16.80–20.01	14.19 ± 0.44	12.86–15.54	77.68
	Girls	17.63 ± 0.61	15.89–19.41	13.61 ± 0.49	12.41–15.19	77.67
7	Boys	18.43 ± 0.63	16.77–19.63	14.32 ± 0.45	12.95–15.75	77.67
	Girls	17.83 ± 0.61	15.89–19.61	13.81 ± 0.47	12.73–15.37	77.70
8	Boys	18.55 ± 0.68	16.82–20.78	14.42 ± 0.67	12.73–16.17	77.77
	Girls	17.91 ± 0.58	16.02–19.58	13.87 ± 0.46	12.74–15.36	77.74
9	Boys	18.65 ± 0.67	17.03–20.77	14.49 ± 0.51	12.89–16.21	77.72
	Girls	18.04 ± 0.64	15.96–19.64	14.01 ± 0.45	12.85–15.35	77.69
10	Boys	18.75 ± 0.66	17.14–20.86	14.55 ± 0.51	13.09–16.21	77.56
	Girls	18.10 ± 0.63	15.97–19.93	14.06 ± 0.44	12.96–15.44	77.60
11	Boys	18.92 ± 0.69	17.21–20.99	14.61 ± 0.58	13.02–16.28	77.22
	Girls	18.24 ± 0.50	16.50–19.90	14.17 ± 0.44	12.96–15.54	77.69
12	Boys	19.07 ± 0.66	17.34–21.06	14.76 ± 0.51	13.29–16.41	77.69
	Girls	18.28 ± 0.55	16.45–19.85	14.28 ± 0.44	13.16–15.54	78.12
13	Boys	19.29 ± 0.64	17.46–21.34	14.87 ± 0.46	13.54–16.36	77.07
	Girls	18.51 ± 0.59	16.41–20.19	14.36 ± 0.50	13.10–15.90	77.09
14	Boys	19.43 ± 0.61	15.08–17.92	15.01 ± 0.56	14.00–16.76	77.25
	Girls	18.59 ± 0.61	14.12–16.98	14.40 ± 0.52	13.60–15.92	77.46
15	Boys	19.52 ± 0.63	15.99–18.21	15.12 ± 0.57	14.20–16.77	77.46
	Girls	18.66 ± 0.65	14.79–18.01	14.44 ± 0.52	13.70–15.92	77.39

^1^ M—mean; SD—standard deviation; min—minimal value; max—maximal value.

**Table 5:** Characteristics of facial length, facial width, and facial index variations for children of different ages.

Age, Years	Gender	Facial Length, cm ^1^		Facial Width, cm ^1^		Facial Index, %
		M ± SD	Min–Max	M ± SD	Min–Max	
1	Boys	7.52 ± 0.27	6.93–8.37	10.77 ± 0.38	9.82–11.88	69.82
	Girls	7.15 ± 0.44	5.76–8.34	10.15 ± 0.50	8.80–11.70	70.44
2	Boys	8.46 ± 0.50	7.30–10.30	11.03 ± 0.32	10.28–11.92	76.70
	Girls	8.13 ± 0.40	6.80–9.30	10.54 ± 0.40	9.30–11.70	77.14
3	Boys	8.90 ± 0.41	7.69–9.91	11.33 ± 0.37	10.33–12.57	78.55
	Girls	8.55 ± 0.33	7.77–9.43	10.86 ± 0.38	9.72–11.78	78.73
4	Boys	9.06 ± 0.40	7.70–10.00	11.07 ± 0.36	9.84–12.76	81.84
	Girls	8.82 ± 0.35	7.95–9.65	11.29 ± 0.34	9.56–12.44	78.12
5	Boys	9.46 ± 0.25	8.85–10.05	11.67 ± 0.38	10.72–12.98	81.06
	Girls	9.09 ± 0.20	8.50–9.60	11.26 ± 0.37	10.03–12.17	80.73
6	Boys	9.69 ± 0.21	9.09–10.21	11.84 ± 0.35	11.05–13.05	81.84
	Girls	9.36 ± 0.30	8.60–10.20	11.50 ± 0.33	10.47–12.23	81.39
7	Boys	9.89 ± 0.28	9.12–10.58	12.07 ± 0.40	11.10–13.30	81.94
	Girls	9.54 ± 0,32	8.78–10.62	11.67 ± 0.33	10.67–12.63	81.75
8	Boys	10.03 ± 0.35	9.25–10.95	12.23 ± 0.27	11.43–13.27	82.01
	Girls	9.66 ± 0.32	8.98–10.72	11.86 ± 0.30	11.00–12.70	81.45
9	Boys	10.10 ± 0.28	11.22–12.98	12.45 ± 0.36	11.64–13.66	81.13
	Girls	9.76 ± 0.33	8.97–10.93	12.11 ± 0.36	11.14–13.06	80.59
10	Boys	10.22 ± 0.37	9.23–11.17	12.65 ± 0.40	11.60–14.00	80.79
	Girls	10.00 ± 0.32	9.18–10.92	12.28 ± 0.38	11.22–13.28	81.43
11	Boys	10.49 ± 0.39	9.21–11.59	12.98 ± 0.37	11.93–13.97	80.82
	Girls	10.24 ± 0.35	9.25–11.15	12.48 ± 0.43	11.17–13.63	82.05
12	Boys	10.60 ± 0.34	9.66–11.54	13.09 ± 0.36	12.24–14.26	80.98
	Girls	10.36 ± 0.34	9.56–11.24	12.69 ± 0.43	11.37–13.83	81.63
13	Boys	10.85 ± 0.36	9.84–11.66	13.29 ± 0,43	12.37–14.53	81.64
	Girls	10.62 ± 0.35	9.75–11.65	12.85 ± 0.46	11.54–14.06	82.65
14	Boys	11.15 ± 0.41	9.99–12.21	13.62 ± 0.43	12.57–15.03	81.87
	Girls	10.71 ± 0.40	9.70–11.90	13.03 ± 0.43	11.77–14.23	82.20
15	Boys	11.36 ± 0.42	10.28–12.52	13.81 ± 0.40	12.90–15.00	82.26
	Girls	10.79 ± 0.40	9.08–12.10	13.11 ± 0.47	11.83–14.47	82.30

^1^ M—mean; SD—standard deviation; min—minimal value; max—maximal value.

**Table 6:** Frequency of head shape types among children of various ages.

Age, Years	Dolichocephaly n ^1^ (%)	Mesocephaly n ^1^ (%)	Brachycephaly n ^1^ (%)	Hyperbrachycephaly n ^1^ (%)
1	134 (36.93)	175 (48.20)	49 (13.49)	17 (4.68)
2	133 (48.36)	117 (42.58)	27 (9.82)	7 (2.54)
3	123 (45.39)	124 (45.78)	26 (9.59)	6 (2.21)
4	96 (34.04)	140 (49.65)	28 (9.93)	6 (2.13)
5	83 (30.62)	135 (49.82)	28 (10.33)	6 (2.21)
6	68 (27.76)	134 (54.72)	28 (11.43)	6 (2.45)
7	63 (26.25)	132 (54.97)	24 (10.00)	8 (3.33)
8	57 (24.25)	130 (55.30)	25 (10.64)	7 (2.98)
9	58 (27.49)	113 (53.60)	25 (11.86)	7 (3.32)
10	57 (27.40)	111 (53.32)	22 (10.57)	9 (4.32)
11	51 (25.89)	107 (54.34)	28 (14.21)	5 (2.54)
12	54 (29.67)	101 (55.49)	22 (12.09)	4 (2.20)
13	47 (26.86)	106 (60.57)	19 (10.86)	3 (1.71)
14	47 (29.93)	94 (59.87)	18 (11.46)	3 (1.91)
15	11 (32.35)	17 (50.00)	4 (11.76)	0 (0.00)

^1^ n—number of children.

**Table 7:** Frequency of face shape types among children of various ages.

Age, Years	Hyper- Euryprosopic n^1^ (%)	Euryprosopic n ^1^ (%)	Mesoprosopic n ^1^ (%)	Leptoprosopic n ^1^ (%)	Hyper- Leptoprosopic n ^1^ (%)
1	240 (64.00)	126 (33.60)	9 (2.40)	0 (0.00)	0 (0.00)
2	213 (75.00)	60 (21.13)	11 (3.87)	0 (0.00)	0 (0.00)
3	181 (64.87)	85 (30.47)	13 (4.66)	0 (0.00)	0 (0.00)
4	140 (51.85)	111 (41.11)	17 (6.30)	1 (0.37)	1 (0.37)
5	103 (40.87)	126 (50.00)	23 (9.13)	0 (0.00)	0 (0.00)
6	82 (34.60)	122 (51.48)	33 (13.92)	0 (0.00)	0 (0.00)
7	65 (28.63)	130 (57.27)	31 (13.66)	1 (0.44)	0 (0.00)
8	67 (30.59)	121 (55.25)	30 (13.70)	1 (0.46)	0 (0.00)
9	77 (37.93)	101 (49.75)	22 (10.84)	3 (1.48)	0 (0.00)
10	66 (33.17)	98 (49.25)	30 (15.08)	4 (2.01)	1 (0.50)
11	68 (35.60)	91 (47.64)	27 (14.14)	4 (2.09)	1 (0.52)
12	66 (36.46)	84 (46.41)	27 (14.92)	3 (1.66)	1 (0.55)
13	68 (38.86)	80 (45.71)	26 (14.86)	1 (0.57)	0 (0.00)
14	58 (35.37)	79 (48.17)	24 (14.63)	2 (1.22)	1 (0.61)
15	4 (12.50)	23 (71.88)	4 (12.50)	1 (3.13)	0 (0.00)

^1^ n—number of children.

**Table 8:** Correlation between the facial index and cephalic index in different age groups.

Age, Years	r_s_ (95% Confidence Interval)	Interpretation	*p*-Value ^1^
1	−0.250 (−0.345–(−0.149))	weak	<0.001 ***
2	−0.183 (−0.297–(−0.065))	weak	<0.002 **
3	−0.306 (−0.411–(−0.192))	moderate	<0.001 ***
4	−0.285 (−0.394–(−0.168))	weak	<0.001 ***
5	−0.331 (−0.440–(−0.213))	moderate	<0.001 ***
6	−0.268 (−0.385–(−0.141))	weak	<0.001 ***
7	−0.266 (−0.386–(−0.136))	weak	<0.001 ***
8	−0.263 (−0.386–(−0.131))	weak	<0.001 ***
9	−0.218 (−0.349–(−0.076))	weak	<0.002 **
10	−0.217 (−0.349–(−0.076))	weak	<0.002 **
11	−0.208 (−0.343–(−0.063))	weak	<0.004 **
12	−0.214 (−0.353–(0.066))	weak	<0.004 **
13	−0.252 (−0.390–(−0.103))	weak	<0.001 ***
14	−0.198 (−0.346–(−0.041))	does not apply	0.320
15	−0.145 (−0.478–0.225)	does not apply	0.430

^1^ ** *p* < 0.01; *** *p* < 0.001 statistical significance (Spearman correlation coefficient); r_s_ (95% confidence interval of rho).

**Table 9:** Descriptive statistics of the facial index and cephalic index in different age groups.

Age, Years	Facial Index ^1^ M ± SD	Cephalic Index ^1^ M ± SD
1	70.13 ± 0.38	77.70 ± 0.53
2	76.92 ± 0.32	76.61 ± 0.48
3	78.64 ± 0.37	76.49 ± 0.53
4	79.98 ± 0.36	76.62 ± 0.53
5	80.90 ± 0.38	77.40 ± 0.53
6	81.62 ± 0.35	77.68 ± 0.54
7	81.85 ± 0.40	77.69 ± 0.54
8	81.73 ± 0.27	77.76 ± 0.56
9	80.86 ± 0.36	77.71 ± 0.57
10	81.11 ± 0.40	77.58 ± 0.57
11	81.44 ± 0.37	77.46 ± 0.59
12	81.31 ± 0.36	77.91 ± 0.60
13	82.15 ± 0.43	77.08 ± 0.60
14	82.04 ± 0.43	77.36 ± 0.61
15	82.28 ± 0.40	77.43 ± 0.61

^1^ M—mean; SD—standard deviation.

**Table 10:** Distribution of facial types across different cephalic index categories.

Facial Type	Dolicho- Cephalic, n ^1^ (%)	Meso- Cephalic, n ^1^ (%)	Brachy- Cephalic, n ^1^ (%)	Hyper- Brachycephalic, n ^1^ (%)	Total, n ^1^ (%)	*p*-Value ^1^
Hypereuryprosopic	440 (13.40)	770 (23.44)	220 (6.70)	66 (2.01)	1496 (45.54)	<0.001 ***
Euryprosopic	533 (16.22)	786 (23.92)	100 (3.04)	18 (0.55)	1437 (43.74)	
Mesoprosopic	100 (3.04)	168 (5.11)	50 (1.520)	9 (0.27)	327 (9.95)	
Leptoprosopic	8 (0.24)	10 (0.30)	2 (0.06)	1 (0.03)	21 (0.64)	
Hyperleptoprosopic	2 (0.06)	2 (0.06)	1 (0.03)	0 (0)	5 (0.15)	
Total, n ^1^ (%)	1083 (32.94)	1736 (52.85)	373 (11.36)	94 (2.86)	3286 (100)	

^1^ n—number of cases; *** *p* < 0.001 statistical significance (Pearson Chi-square test).

## Data Availability

The data presented in this study are available upon request from the corresponding author due to ethical reasons.
